# Resistance to colistin and fosfomycin in extended-spectrum beta-lactamase-producing *Escherichia coli*

**DOI:** 10.17843/rpmesp.2025.423.14482

**Published:** 2025-09-25

**Authors:** Ana Paula Roldan-Galarreta, Maria Fernanda Arce-Servat, Franco Arancivia-Neira, Edgar Gonzales-Escalante

**Affiliations:** 1 Facultad de Medicina Humana, Universidad de Piura, Lima, Peru. Universidad de Piura Facultad de Medicina Humana Universidad de Piura Lima Peru

**Keywords:** Drug resistance, Microbial, Escherichia coli, Chickens, Fosfomycin, Colistin

## Abstract

The objective of our study was to determine the resistance to colistin and fosfomycin of extended-spectrum beta-lactamase-producing *Escherichia coli* (Ec-BLEE). We conducted a cross-sectional descriptive study in which we analyzed 79 isolates of Ec-BLEE obtained from a previous study involving 300 chicken cloacal swab samples. We processed the isolates in the Microbiology and Immunology Laboratory of the Faculty of Medicine of the Universidad de Piura, Lima-Peru. We determined the sensitivity to colistin with the Colistin-AgarSpot detection medium and the sensitivity to fosfomycin by antibiogram according to the CLSI guidelines. We searched for the *mcr-1* and *fosA3* genes through PCR. We found a resistance to colistin in 17,7% and to fosfomycin in 77% of the samples. We did not detect the *mcr-1* gene in the colistin-resistant isolates. The *fosA3* gene was present in 100% of the fosfomycin resistant isolates.

## INTRODUCTION

The “One Health” approach, promoted by the World Health Organization (WHO), highlights the interconnection between human, animal, and environmental health, and recognizes that antimicrobial resistance (AMR) affects these three domains ^1^. AMR is a growing problem due to the spread of microorganisms between communities, hospitals, food, and the environment, affecting human and animal health ^1^. It is estimated that two out of every five deaths are associated with resistant bacteria, and *Escherichia coli* (*E. coli*) is the second leading cause of these deaths ^2^. One of the main resistance mechanisms of *E. coli* is the production of extended-spectrum beta-lactamases (ESBLs), enzymes that hydrolyze and inactivate beta-lactam antibiotics ^3^.

In Peru, the poultry industry is the main source of animal protein, with an average consumption of 52.3 kg per capita in 2023 ^4^. The use of antimicrobials for therapeutic, prophylactic, metaphylactic purposes, and as growth promoters in the poultry industry fosters AMR ^1^. The administration of sub-therapeutic doses in chickens predisposes the emergence of AMR in animals and its possible transmission to humans ^1^.

Colistin, an antibiotic used as a last-line treatment for multidrug-resistant (MDR) infections, has *mcr-1* as its main resistance gene, capable of modifying the lipid A of the cell membrane ^5^. In 2019, the Ministry of Health of Peru banned the use of colistin and its derivatives for veterinary purposes or in livestock production ^6^. Fosfomycin, formerly used for uncomplicated urinary tract infections, has gained recognition for its activity against both gram-positive and gram-negative bacteria ^5^. The *fosA3* gene encodes a metalloenzyme that inactivates fosfomycin and is transmissible among *Enterobacterales* via mobile genetic elements, causing a more rapid dissemination of resistance ^5^^,^^7^.

In Peru, as in other countries, AMR challenges public health ^8^^-^^14^. Epidemiological surveillance of AMR in food-producing animals is limited to identifying antimicrobial susceptibility; however, we consider it necessary to perform a molecular analysis of resistance genes and other characteristics of resistant isolates. For this reason, the objective of our study was to determine the presence of resistance to colistin and fosfomycin in ESBL-producing *E. coli* (ESBL-EC) isolates from chickens for human consumption in Lima, Peru.

**KEY MESSAGES**

Motivation for the study. Given the growing problem of antimicrobial resistance that threatens the “One Health” approach, we analyzed 79 isolates of extended-spectrum beta-lactamase-producing *Escherichia coli* and their resistance to last-resort antibiotics.

Main findings. We observed high resistance to fosfomycin (77%); all resistant isolates carried the fosA3 gene, and the gene was transferred in all conjugation assays. Resistance to colistin was 17.7%, and we did not detect the mcr-1 gene in any sample.

Implications. These findings underscore the importance of monitoring antimicrobial resistance in bacteria present in avian-derived foods to prevent its spread to humans.

## THE STUDY

We conducted a descriptive cross-sectional study of 79 consecutive non-duplicate ESBL-EC isolates recovered from chicken cloacae; isolates were obtained from project PI2206 of the School of Medicine at the Universidad de Piura (FMH-UDEP) titled Bacterial resistance in *Enterobacterales* recovered from chickens intended for human consumption. The primary study collected 300 samples from cloacal swabs of chickens from two collection centers (located in the districts of Ate and San Martín de Porres, both in Lima). The samples were cultured on CHROMagar™ ESBL (bioMérieux, France), a selective medium for detecting gram-negative ESBL-producing bacteria, and 79 ESBL-EC isolates were recovered, which were used in the present study. The isolates were stored at -4 °C in the Microbiology and Immunology Laboratory of the FMH-UDEP, in Lima, Peru. The procedures were performed between January and July 2024 in the laboratory.

The 79 isolates were streaked on McConkey agar and subsequently incubated at 37 °C for 24 hours to reactivate them and verify their viability and purity. We determined colistin susceptibility using the Colistin-AgarSpot detection medium, developed by the Antimicrobial Service of the National Institute of Infectious Diseases of the National Administration of Laboratories and Health Institutes of Argentina, with a colistin concentration of 3 µg/mL ^15^. It was considered resistant if one or more ESBL-EC colonies grew and sensitive if no colony grew ^15^.

The susceptibility of ESBL-EC to fosfomycin was determined by the disk diffusion method, using the guidelines of the CLSI (Clinical and Laboratory Standards Institute) ^16^. It was classified as resistant and sensitive if it had an inhibition zone of less than 12 mm and greater than 16 mm, respectively ^16^.

Molecular detection was performed in the laboratory. We used total DNA as a template. We used the procedures described by Lescat *et al.*^17^ for the *mcr-1* gene. We used 1 μL of *forward*: 5´-ATGCCAGTTTCTTTCGCGTG-3´ and *reverse*: 5´-TCGGCAAATTGCGCTTTTGGC-3´ primers, with 2 μL of 10X PCR Buffer (100 mM potassium chloride, 100 mM Tris hydrochloride, 20 mM magnesium chloride), 1 µL of 10 mM deoxynucleotide triphosphate (dNTPs), 0.2 µL of 5 U/µL Taq polymerase (CavBio, Peru), and 2 µL of DNA for a final volume of 20 µL. The conditions were as follows: denaturation at 94 °C for 4 min; alignment at 94 °C for 5 seconds and 59 °C for 20 seconds for 30 cycles each, and elongation at 72 °C for 5 minutes. For the *fosA3* gene, we used the procedures described by Hou et al. (7). We used 1.5 µL of *forward*: 5’-GCGTCAAGCCTGGCATTT-3’ and *reverse*: 5’-TCAATCCGGACTCACTGCAG-3’ primers with 2.5 µL of 10X PCR Buffer, 0.5 µL of 10 mM dNTPs, 0.2 µL of 5 U/µL Taq polymerase, and 2 µL of DNA in a final volume of 25 µL. The conditions were as follows: denaturation at 95 °C for 4 minutes; alignment at 95 °C for 30 seconds, 52 °C for 30 seconds, and 72 °C for 30 seconds for 30 cycles, and a final elongation at 72 °C for 5 minutes.

We analyzed the PCR products with an electrophoresis run on a 1.5% agarose gel with *SYBR Safe* in 1X TAE (Tris-Acetate-EDTA) buffer for 30 minutes at 100 volts. Subsequently, we photographed the gel in the gel doc system.

We performed conjugation assays to determine the mechanisms of plasmid-mediated resistance transfer for colistin and fosfomycin. We used the *E. coli* J53 strain (resistant to sodium azide) as the recipient and the fosfomycin- or colistin-resistant strains as donors. We mixed 0.5 mL of the cultures in Müller Hinton broth of the donor and recipient strains in a test tube. Then, we incubated the mixtures for 3 hours at 37 °C, centrifuged for 2 minutes at 17,000 g in a Sorvall™ Legend™ Micro 17 microcentrifuge, and suspended the pellet in 100 μL of Luria Bertani broth (LBA). We selected the transconjugants on LBA medium containing 128 μg/mL fosfomycin and 150 μg/mL sodium azide to evaluate the transfer of *fosA3*, and 2 μg/mL colistin and 150 μg/mL sodium azide to evaluate the transfer of *mcr-1*, incubating at 37 °C for 24 hours ^18^.

We stored the data in a Microsoft Excel database and performed a descriptive analysis of the absolute and relative frequencies of the colistin- and fosfomycin-resistant isolates.

We received approval from the Ethics Committee of the FMH-UDEP with file N° REMED06202328. The researchers of the primary study gave authorization to perform the sub-analysis.

## FINDINGS

With the Colistin-AgarSpot method, 14 isolates (17.7%) showed resistance to colistin. Using the disk diffusion method, 61 isolates (77.2%) showed resistance to fosfomycin. We did not detect the presence of the *mcr-1* gene in any colistin-resistant isolate with molecular characterization using specific primers. All fosfomycin-resistant isolates carried the *fosA3* gene.

We performed horizontal plasmid transfer by conjugation assays on 12 colistin-resistant and 11 fosfomycin-resistant isolates; all selected according to their susceptibility profile and place of origin. We found successful transfer only in the fosfomycin-resistant isolates.

We verified the transconjugants (TC) by determining the susceptibility to fosfomycin and other antibiotics (Table 1), where the susceptibility tests for the donor cells and TCs of the 11 isolates confirmed the transfer of *fosA3*-carrying plasmids by conjugation, resulting in a persistence of non-susceptible values to fosfomycin. Finally, we confirmed that there was a successful transfer of *fosA3* via PCR.

**Table 1:** Susceptibility profile of fosfomycin-resistant ESBL-producing *E. coli* and their respective transconjugants.

Isolates	Inhibition zones (mm)*											
	FOS	CIP	STX	CLO	GE	AK	CAZ	CTX	FEP	FOX	IPM	MEM
A8	6	6	6	6	6	25	18	10	20	14	34	34
TC A8	6	30	32	27	6	25	22	9	15	26	34	32
A20	6	20	6	6	6	25	22	12	22	26	32	32
TC A20	14	40	31	25	24	23	22	14	20	30	32	34
A39	6	6	6	6	6	24	20	12	20	22	32	32
TC A39	6	30	6	6	6	22	23	14	25	24	24	29
A75	6	6	6	6	6	25	18	12	18	32	32	32
TC A75	14	40	31	32	24	24	27	16	20	31	33	34
A76	6	6	6	6	6	21	20	12	20	22	34	34
TC A76	6	34	6	7	8	24	27	16	24	31	33	33
A80	6	6	6	6	6	18	22	12	22	20	28	28
TC A80	6	44	29	28	25	26	18	20	14	30	24	30
A96	6	20	6	6	6	25	22	14	20	24	30	30
TC A96	15	40	32	24	27	25	20	16	20	18	32	32
A132	6	26	6	2	6	20	20	6	24	23	30	30
TC A132	6	40	6	6	6	25	20	14	20	28	30	34
A139	6	32	6	2	6	21	26	16	26	26	30	30
TC A139	6	40	30	22	6	26	28	12	18	26	30	30
A140	6	27	6	2	6	20	26	14	22	24	28	28
TC A140	14	42	32	24	26	25	18	12	18	30	30	30
A142	6	6	6	2	6	22	18	6	12	23	28	28
TC A142	6	40	31	6	28	26	18	12	22	32	34	30

TC: transconjugants, CIP: ciprofloxacin, STX: sulfamethoxazole/trimethoprim, CLO: chloramphenicol, GE: gentamicin, FOS: fosfomycin, AK: amikacin, CAZ: ceftazidime, CTX: cefotaxime, FEP: cefepime, FOX: cefoxitin, IMP: imipenem, MEM: meropenem.

* The inhibition zone values correspond to one observation per isolate.

## DISCUSSION

MDR *E. coli* is a global health threat with documented cases in different niches, and it represents a potential risk of transmission to humans ^8^^-^^14^^,^^19^. We found a colistin resistance of 17.7%, a value lower than that reported in Brazil, where a prevalence of 30% was observed in 78 ESBL-EC isolates ^8^. In Bangladesh, a prevalence of 78% was found, although these bacteria were not ESBL-producers ^9^. For fosfomycin, we report a resistance of 77.2% ^8^, higher than the 40% reported in Brazil in *E. coli* isolates from a farm that used fosfomycin compared to 5% in another farm that did not ^8^. According to One Health, this becomes relevant as it suggests a possible link between the use of antimicrobials in the poultry industry and the production of resistant isolates ^1^.

We did not detect the *mcr-1* gene in any colistin-resistant isolate, a result similar to that reported in Brazil, where it was found in 1 out of 60 ESBL-EC isolates in backyard chickens ^8^. This contrasts with a Peruvian study that found *mcr-1* in 21.3% of 197 chicken samples collected between 2019 and 2020 ^10^, prior to the implementation of the law; whereas our isolates were recovered in 2022, after the implementation. Despite the ban on the use of colistin in animals in Peru since December 2019, our results show the persistence of resistance to this antimicrobial.

In China, a similar measure reduced resistance from 11.4% to 0.9% in two years after its implementation ^12^. The presence of colistin-resistant ESBL-EC in our study could suggest that this measure was not sufficient to eliminate them, but we cannot determine its direct relationship with the implemented policy. Although the main mechanism of colistin resistance is the *mcr-1* gene, the colistin resistance found in our study could be due to mutations in *pmr* (e.g., pmrB Y84C, PI4L or H81R) and *pho* (e.g., pho I44L) genes ^20^ that regulate the expression of lipid A, mutations that were not the subject of analysis in this study ^5^.

We detected the *fosA3* gene in 77.2% of the ESBL-EC isolates, higher than what was found in Brazil, which reported its presence in 68% of 507 ESBL-EC isolates from one-day-old chick samples ^13^. Another study in Brazil with fosfomycin-resistant ESBL-EC showed a prevalence of the *fosA3* gene of 73% in 26 isolates from a farm that used fosfomycin, while the gene was not detected in three isolates from the farm that did not use it ^8^. A possible explanation could be the use of fosfomycin on poultry farms in Peru, which could be related to the prevalence of the *fosA3* gene and fosfomycin resistance in these environments ^8^. Likewise, we demonstrated the transfer of the *fosA3* gene in 100% of the conjugation assays, similar to a study in China, with 98.9% of isolates positive after the assay ^14^ and another in Brazil with 100% ^8^, although the latter only performed it on 2 isolates. The high resistance rates may be because *fosA3* can be co-transferred with other resistance genes, such as *blaCTX-M*, which confers resistance to beta-lactams ^7^.

One of the main limitations was the number of samples, as we only worked with two collection centers. However, this study represents a first descriptive approximation of the phenomenon. Another limitation was that we only searched for the *mcr-1* and *fosA3* genes, as they are the most frequent, although other less common resistance mechanisms exist that were not evaluated. The original study selected only ESBL-EC isolates from the 300 samples, without considering resistance to colistin and fosfomycin. This could underestimate the reported resistance percentages, as some isolates that might be resistant were not analyzed because they were not ESBL-EC.

Finally, our results do not encompass the national situation, but they offer valuable evidence on the resistance of ESBL-EC and highlight the urgency of investigating other resistance mechanisms to colistin and fosfomycin. In conclusion, we detected the presence of ESBL-EC resistant to these antibiotics, constituting a potential risk to human health. In line with the One Health approach, it is imperative to strengthen AMR surveillance and control the indiscriminate use of antibiotics in food-producing animals, as it constitutes a threat to public health that requires multi-sectoral interventions.
