# Supplementary material for: Resistance to colistin and fosfomycin in extended-spectrum beta-lactamase-producing Escherichia coli
Source: Rev Peru Med Exp Salud Publica. 2025 Sep 25;42(3):307–11. doi: 10.17843/rpmesp.2025.423.14482 (PMC12679967; doi:10.17843/rpmesp.2025.423.14482)
Supplement: Translation

# Translation: Resistencia a colistina y fosfomicina en aislamientos de *Escherichia coli* productora de betalactamasas de espectro extendido provenientes de pollos de consumo humano

**Authors:** Ana Paula Roldan-Galarreta, Maria Fernanda Arce-Servat, Franco Arancivia-Neira, Edgar Gonzales-Escalante

**Affiliations:** 1 Facultad de Medicina Humana, Universidad de Piura, Lima, Perú.

**Keywords:** Farmacorresistencia Microbiana, Escherichia coli, Pollos, Fosfomicina, Colistina

## Abstract

El objetivo del estudio fue determinar la presencia de resistencia a colistina y fosfomicina en aislamientos de *Escherichia coli* productoras de betalactamasas de espectro extendido (Ec-BLEE) provenientes de pollos de consumo humano. Realizamos un estudio descriptivo transversal y analizamos 79 aislamientos de Ec-BLEE, provenientes de un estudio previo de 300 muestras de hisopado cloacal de pollo. Los aislamientos fueron procesados en el laboratorio de la Facultad de Medicina de la Universidad de Piura. La sensibilidad a colistina se determinó con el medio de detección Colistin-AgarSpot y a fosfomicina mediante antibiograma según los lineamientos de la guía CLSI. Buscamos los genes *mcr-1* y *fosA3* a través de PCR. Encontramos resistencia a colistina en el 17,7% y a fosfomicina en el 77% de las muestras. El gen *mcr-1* no estuvo presente en los aislamientos resistentes a colistina y *fosA3* estuvo presente en 100% de los aislamientos resistentes a fosfomicina.

## INTRODUCCIÓN

El enfoque «Una sola salud» (One Health), promovido por la Organización Mundial de la Salud (OMS), resalta la interconexión entre la salud humana, animal y ambiental, y reconoce que la resistencia antimicrobiana (RAM) afecta a estos 3 ámbitos ^1^. La RAM es un problema creciente por la propagación de microorganismos entre comunidades, hospitales, alimentos y el ambiente, afectando la salud humana y animal ^1^. Se estima que dos de cada cinco muertes están asociadas a bacterias resistentes y *Escherichia coli (E. coli)* es el segundo responsable de estas muertes ^2^. Uno de los principales mecanismos de resistencia de *E. coli* es la producción de betalactamasas de espectro extendido (BLEE), enzimas que hidrolizan antibióticos betalactámicos y los inactivan ^3^.

En Perú, la industria avícola es la principal fuente de proteína animal, con un consumo promedio de 52,3 kg por habitante en el 2023 ^4^. El uso de antimicrobianos con fines terapéuticos, profilácticos, metafilácticos y como promotores de crecimiento en la industria avícola favorece la RAM ^1^. La administración de dosis subterapéuticas en pollos predispone la aparición de RAM en animales y posible transmisión hacia humanos ^1^.

La colistina, antibiótico utilizado como última línea en el tratamiento de infecciones multidrogorresistentes (MDR), tiene como principal gen de resistencia a *mcr-1*, capaz de modificar el lípido A de la membrana celular ^5^. En 2019, el Ministerio de Salud del Perú prohibió el uso de colistina y derivados con fines veterinarios o en producción ganadera ^6^. La fosfomicina, antiguamente utilizada para infecciones urinarias no complicadas, ha ganado reconocimiento por su actividad frente a bacterias grampositivas y gramnegativas ^5^. El gen *fosA3* codifica una metaloenzima que inactiva fosfomicina y es transmisible entre *Enterobacterales* mediante elementos genéticos móviles, lo que causa una diseminación más rápida de resistencia ^5^^,^^7^.

En Perú, como en otros países, la RAM desafía a la salud pública ^8^^-^^14^. La vigilancia epidemiológica de la RAM en animales de consumo se limita a la identificación de la sensibilidad antimicrobiana, sin embargo, consideramos necesario realizar un análisis molecular de los genes de resistencia y otras características de los aislamientos resistentes. Por esta razón, el objetivo de nuestro estudio fue determinar la presencia de resistencia a colistina y fosfomicina en aislamientos de *E. coli* productoras de BLEE (Ec-BLEE) provenientes de pollos de consumo humano Lima, Perú.

**MENSAJE CLAVE**

Motivación para el estudio. Ante el creciente problema de resistencia antimicrobiana que amenaza el enfoque de «Una sola salud», analizamos 79 aislamientos de *Escherichia coli* productoras de betalactamasas de espectro extendido y su resistencia frente a antibióticos de último recurso.

Principales hallazgos. Observamos una alta resistencia a fosfomicina (77%), todos los aislamientos resistentes presentaban el gen fosA3 y el gen fue transferido en todos los ensayos de conjugación. La resistencia a colistina fue del 17,7%, y no detectamos el gen mcr-1 en ninguna muestra.

Implicancias. Estos hallazgos subrayan la importancia de monitorear la resistencia antimicrobiana en bacterias presentes en alimentos de origen aviar para prevenir su propagación a humanos.

## EL ESTUDIO

Realizamos un estudio transversal descriptivo de 79 aislamientos consecutivos no repetidos de Ec-BLEE recuperadas de cloacas de pollo; aislamientos obtenidos del proyecto PI2206 de la Facultad de Medicina de la Universidad de Piura (FMH-UDEP) denominado Resistencia bacteriana en *Enterobacterales* recuperadas de pollos destinados al consumo humano; Estudio primario que recolectó 300 muestras de hisopados cloacales de pollo de dos centros de acopio (Ubicados en los distritos de Ate y San Martín de Porres, ambos en Lima). Las muestras fueron sembradas en CHROMagar^TM^ ESBL (bioMerieux, Francia), medio selectivo para detectar bacterias gramnegativas productoras de BLEE, y se recuperaron 79 aislamientos Ec-BLEE los que fueron utilizados en el presente estudio. Los aislamientos fueron conservados a -4 °C en el Laboratorio de Microbiología e Inmunología de la FMH-UDEP, en Lima-Perú. Los procedimientos fueron realizados entre enero y julio del 2024 en el laboratorio.

Los 79 aislamientos fueron sembrados en agar McConkey y posteriormente incubados a 37 °C por 24 horas para reactivarlos y verificar su viabilidad y pureza. Determinamos la sensibilidad a colistina utilizando el medio de detección Colistin-AgarSpot, desarrollado por el Servicio Antimicrobiano del Instituto Nacional de Enfermedades Infecciosas de la Administración Nacional de Laboratorios e Institutos de Salud de Argentina, con concentración de 3 µg/mL de colistina ^15^. Considerándose resistente si crecieron una o más colonias de Ec-BLEE y sensible si no creció colonia alguna ^15^.

La sensibilidad de Ec-BLEE a fosfomicina se determinó mediante antibiograma con discos de difusión, utilizando los lineamientos de la guía CLSI *(Clinical and Laboratory Standards Institute)*^16^. Se clasificó resistente y sensible si presentaba un halo de inhibición menor a 12 mm y mayor a 16 mm, respectivamente ^16^.

La detección molecular se realizó en el laboratorio. Utilizamos ADN total como molde. Utilizamos los procedimiento descritos por Lescat *et al*^17^ para el gen *mcr-1*. Utilizamos 1 μL de cebadores *forward:* 5´-ATGCCAGTTTCTTTCGCGTG-3´ y *reverse:* 5′-TCGGCAAATTGCGCTTTTGGC-3, con 2 μL de Buffer de PCR 10X (100 mM de cloruro de potasio, 100 mM de clorhidrato de Tris, 20 mM de cloruro de magnesio 2), 1 µL de trifosfato de desoxinucleótido (dNTPs) 10 mM, 0,2 µL de Taq polimerasa 5 U/µL (CavBio, Perú) y 2 µL de ADN para un volumen final de 20 µL. Las condiciones fueron las siguientes: desnaturalización a 94 °C durante 4 min; alineamiento a 94 °C por 5 segundos y 59 °C durante 20 segundos por 30 ciclos cada uno y elongación a 72 °C durante 5 minutos. Para el gen *fosA3* utilizamos los procedimientos descritos por Hou *et al*^7^. Utilizamos 1,5 µL de cebadores *forward:* 5’-GCGTCAAGCCTGGCATTT-3’ y *reverse*: 5’-TCAATCCGGACTCACTGCAG-3’ con 2,5 µL de Buffer de PCR 10X, 0,5 µL de dNTPs 10 mM, 0,2 µL de Taq polimerasa 5 U/µL y 2 µL de ADN en un volumen final de 25 µL. Las condiciones fueron las siguientes: desnaturalización a 95 °C durante 4 minutos; alineamiento a 95 °C durante 30 segundos, 52 °C durante 30 segundos y 72 °C durante 30 segundos durante 30 ciclos y una elongación final a 72 °C durante 5 minutos.

Analizamos los productos de la PCR con una corrida de electroforesis en un medio de gel agarosa al 1,5% con *SYBR Safe* en buffer TAE (Tris-Acetate-EDTA) 1X por 30 minutos a 100 voltios. Posteriormente, fotografiamos el gel en el fotodocumentador.

Realizamos ensayos de conjugación para determinar los mecanismos de transferencia de resistencia a través de plásmidos a colistina y fosfomicina. Utilizamos la cepa *E. coli* J53 (resistente a azida de sodio) como receptora y las cepas resistentes a fosfomicina o colistina como dadoras. Mezclamos 0,5 mL de los cultivos en caldo Müller Hinton de la cepa dadora y de la receptora en un tubo de ensayo. Luego, incubamos las mezclas por 3 horas a 37 °C, centrifugamos por 2 minutos a 17 000 g en una microcentrífuga Sorvall^TM^ Legend^TM^ Micro 17 y suspendimos el sedimento en 100 μL de caldo Luria Bertani (LBA). Seleccionamos las transconjugantes en medio LBA que contenía fosfomicina 128 μg/mL y azida de sodio 150 μg/mL para evaluar la transferencia de *fosA3*, y colistina 2 μg/mL y azida de sodio 150 μg/mL para evaluar la transferencia de *mcr-1*, incubando a 37 °C por 24 horas ^18^.

Almacenamos los datos en una base en *Microsoft Excel*, realizamos un análisis descriptivo de las frecuencias absolutas y relativas de los aislamientos resistentes a colistina y fosfomicina.

Recibimos la aprobación del Comité de Ética de la FMH-UDEP con expediente N° REMED06202328. Los investigadores del estudio primario dieron autorización para realizar el subanálisis.

## HALLAZGOS

Con el método Colistin-AgarSpot, 14 aislamientos (17,7%) mostraron resistencia a colistina. Mediante el método de disco de difusión, 61 aislamientos (77,2%) presentaron resistencia a fosfomicina. No detectamos la presencia del gen *mcr-1* en ningún aislamiento resistente a colistina con la caracterización molecular utilizando cebadores específicos. Todos los aislamientos resistentes a fosfomicina presentaron el gen *fosA3*.

Realizamos la transferencia horizontal de plásmidos por ensayos de conjugación de 12 aislamientos resistentes a colistina y 11 a fosfomicina; todos seleccionados de acuerdo a su perfil de sensibilidad y lugar de procedencia. Encontramos una transferencia exitosa sólo en los aislamientos resistentes a fosfomicina.

Verificamos las transconjugantes (TC) mediante la determinación de sensibilidad de fosfomicina y otros antibióticos (Tabla 1) donde los ensayos de sensibilidad para las células dadoras y TC de los 11 aislamientos confirmaron transferencia de los plásmidos portadores de *fosA3* por conjugación, resultando en una persistencia de valores no sensibles a fosfomicina. Finalmente, confirmamos que si hubo una transferencia exitosa de *fosA3* a través de PCR.

**Tabla 1:** Perfil de sensibilidad de las *E. coli* productoras de BLEE resistentes a fosfomicina y sus respectivas transconjugantes

Aislamientos	Halos de inhibición (mm)^a^											
	FOS	CIP	STX	CLO	GE	AK	CAZ	CTX	FEP	FOX	IPM	MEM
A8	6	6	6	6	6	25	18	10	20	14	34	34
TC A8	6	30	32	27	6	25	22	9	15	26	34	32
A20	6	20	6	6	6	25	22	12	22	26	32	32
TC A20	14	40	31	25	24	23	22	14	20	30	32	34
A39	6	6	6	6	6	24	20	12	20	22	32	32
TC A39	6	30	6	6	6	22	23	14	25	24	24	29
A75	6	6	6	6	6	25	18	12	18	32	32	32
TC A75	14	40	31	32	24	24	27	16	20	31	33	34
A76	6	6	6	6	6	21	20	12	20	22	34	34
TC A76	6	34	6	7	8	24	27	16	24	31	33	33
A80	6	6	6	6	6	18	22	12	22	20	28	28
TC A80	6	44	29	28	25	26	18	20	14	30	24	30
A96	6	20	6	6	6	25	22	14	20	24	30	30
TC A96	15	40	32	24	27	25	20	16	20	18	32	32
A132	6	26	6	2	6	20	20	6	24	23	30	30
TC A132	6	40	6	6	6	25	20	14	20	28	30	34
A139	6	32	6	2	6	21	26	16	26	26	30	30
TC A139	6	40	30	22	6	26	28	12	18	26	30	30
A140	6	27	6	2	6	20	26	14	22	24	28	28
TC A140	14	42	32	24	26	25	18	12	18	30	30	30
A142	6	6	6	2	6	22	18	6	12	23	28	28
TC A142	6	40	31	6	28	26	18	12	22	32	34	30

TC: transconjugantes, CIP: ciprofloxacino, STX: sulfametoxazol/trimetoprima, CLO: cloranfenicol, GE: gentamicina, FOS: fosfomicina, AK: amikacina, CAZ: ceftazidima, CTX: cefotaxima, FEP: cefepima, FOX: cefoxitina, IMP: imipenem, MEM: meropenem.

a Los valores de halos de inhibición corresponden a una observación por aislamiento.

## DISCUSIÓN

*E. coli* MDR es una amenaza a la salud mundial con casos documentados en diferentes nichos, y representa un riesgo potencial de transmisión a humanos ^8^^-^^14^^,^^19^. Encontramos una resistencia a colistina de 17,7%, valor inferior al reportado en Brasil, donde se observó una prevalencia de 30% en 78 aislamientos de Ec-BLEE ^8^. En Bangladesh se halló una prevalencia del 78%, aunque, estas bacterias no eran productoras de BLEE ^9^. Para fosfomicina, reportamos una resistencia de 77,2% ^8^, superior al 40% reportado en Brasil en aislamientos de *E. coli* de una granja que utilizaba fosfomicina comparada al 5% en otra granja que no lo utilizaba ^8^. De acuerdo a One Health, esto cobra relevancia ya que, sugiere un posible vínculo entre el uso de antimicrobianos en la industria avícola y la producción de aislamientos resistentes ^1^.

No detectamos el gen *mcr-1* en ningún aislamiento resistente a colistina, resultado similar al reportado en Brasil, donde encontraron este gen en 1/60 aislamientos de Ec-BLEE en pollos de corral ^8^. Esto contrasta con un estudio peruano que halló *mcr-1* en el 21,3% de 197 muestras de pollos; recolectadas entre 2019 y 2020 ^10^, previo a la implementación de la ley; mientras que nuestros aislamientos fueron recuperados en 2022, luego de la implementación. A pesar de la prohibición del uso de colistina en animales en Perú desde diciembre de 2019, nuestros resultados muestran la persistencia de resistencia a este antimicrobiano.

En China, una medida similar redujo la resistencia del 11,4% al 0,9% en dos años tras su implementación ^12^. La presencia de Ec-BLEE resistentes a colistina en nuestro estudio podría sugerir que dicha medida no fue suficiente para eliminarlas, pero no podemos determinar su relación directa con la política implementada. A pesar que el principal mecanismo de resistencia a colistina es el gen *mcr-1*, la resistencia a colistina hallada en nuestro estudio podría deberse a mutaciones en genes *pmr* (p.ej. *pmrB* Y84C, PI4L o H81R) y *pho* (p.ej. *pho* I44L) ^20^ que regulan la expresión del lípido A, mutaciones que no fueron objeto de análisis de este estudio ^5^.

Detectamos el gen *fosA3* en el 77,2% de los aislamientos Ec-BLEE, superior a lo encontrado en Brasil, que reportó la presencia en 68% de 507 aislamiento de Ec-BLEE provenientes de muestras de pollo de un día de vida ^13^*.* Otro estudio en Brasil con Ec-BLEE resistentes a fosfomicina, mostró una prevalencia del gen *fosA3* del 73% en 26 aislamientos de una granja que utilizaba fosfomicina, mientras que no se detectó el gen en tres aislamientos provenientes de la granja que no la utilizaba ^8^. Una posible explicación sería el uso de fosfomicina en las granjas avícolas del Perú, lo cual podría estar relacionado con la prevalencia del gen *fosA3* y la resistencia a fosfomicina en estos entornos ^8^. Asimismo, demostramos la transferencia del gen *fosA3* en el 100% de los ensayos de conjugación, similar a un estudio en China, con 98,9% de aislamientos positivos tras el ensayo ^14^ y otro en Brasil con 100% ^8^, aunque este último solo lo realizó en 2 aislamientos. Las altas tasas de resistencia pueden deberse a que *fosA3* puede co-transferirse con otros genes de resistencia como *bla*_CTX-M_ que otorga la resistencia a betalactámicos ^7^.

Una de las principales limitaciones fue el número de muestras, ya que sólo se trabajó con dos centros de acopio. Sin embargo, este estudio representa una primera aproximación descriptiva al fenómeno. Otra limitación fue que sólo buscamos los genes *mcr-1* y *fosA3,* debido a que son los más frecuentes, aunque existen otros mecanismos de resistencia menos comunes que no fueron evaluados. El estudio original seleccionó sólo aislamientos Ec-BLEE de las 300 muestras, sin considerar la resistencia a colistina y fosfomicina. Esto podría subestimar los porcentajes de resistencia reportados, ya que algunos aislamientos que podrían ser resistentes no fueron analizados por no ser Ec-BLEE.

Finalmente, nuestros resultados no engloban la situación nacional, pero ofrecen evidencia valiosa sobre la resistencia de Ec-BLEE y destacan la urgencia de investigar otros mecanismos de resistencia ante colistina y fosfomicina. En conclusión, detectamos la presencia de Ec-BLEE resistentes a estos antibióticos, constituyendo un riesgo potencial para la salud humana. En línea con el enfoque One Health, es imperante reforzar la vigilancia de la RAM y controlar el uso indiscriminado de antibióticos en animales de consumo humano, debido a que constituye una amenaza para la salud pública que requiere intervenciones multisectoriales.
